# How an Autocrat Dies

**Published:** 1863-12

**Authors:** 


### HALL'S JOURNAL OF HEALTH.

Our Legitimate Scope is almost boundless: for whatever begets pleasurable and harmless feelings, promotes Health; and whatéver induces disagreeable sensations, engenders Disease.

WE AIM TO SHOW HOW DISEASE MAY BE AVOIDED, AND THAT IT IS BEST, WHEN SICKNESS COMES, TO TAKE NO MEDICINE WITHOUT CONSULTING A PHYSICIAN.

Vol. X.] DECEMBER, 1863. [No. 12.

### HOW AN AUTOCRAT DIES.

A HIGH degree of worldly prosperity leads most men, who have been unused to it, to forget God. On the other hand, a high position, as to money or power, continued for generations, often inclines men to seek the solaces of religion. The poor are prone to think that in riches there is happiness. The rich know that riches do not secure happiness, and hence look to religion as the only source left for enduring pleasures, and the hopes of heaven. Hence, most of the greatest of earth's rulers this day, recognize the claims of Christianity as they understand it, and more or less square their lives by its precepts.

The King of Hanover is afflicted with blindness, but we are pleased to learn from the *News of the Churches* that "the eyes of his understanding have for years past been enlightened;" that he is not only a believer in the Lord Jesus, but a confessor of his name, and neither ashamed to own himself a disciple, nor to defend the cause of his Lord before high and low. On the occasion of the laying of the corner-stone of a new church in his capital, he made some remarks which indicated a heart experienced in spiritual things. He said in concluding:

"Furthermore, I entreat the Almighty to permit that the Gospel of his dear Son may be transmitted from this church to all heathen lands, it being my desire and resolve, that henceforth all Hanoverian missionaries shall receive ordination within the walls of this Christ church, which may thus become a well of salvation, not only to its own congregation, but to the nations of the farthest regions of the globe."

The late Nicholas of Russia, the Autocrat of sixty millions of subjects, was one of these, as would seem from incidents occurring in his last and brief illness, the authority being from a source very near the throne.

"Towards night the last glimmer of a chance of recovery disappeared. On being made acquainted with the fact, the Empress, like a Christian wife, went to his bedside promptly, to impart the important intelligence to her dying husband, he being all unconscious that he was even dangerously ill. The Emperor was not prepared for this visit. She bent down over his bed and said to him tenderly and gently." It should be remembered here that, a few days before, while partaking of the Holy Sacrament, a feeling of debility or exhaustion came over the Emperor, so that he could not proceed with it. Under these circumstances, it is wonderful with what adroitness and affectionate delicacy and presence of mind the Empress made use of the fact. "My dear, you were not able to finish your devotions, so as to commune together with us, as on former occasions. Why should you not do it now? You know that for a Christian there is no better medicine, and many have even been recovered by it from their sickness."

"How!" replied the Emperor quickly, "would you have me commune in bed? I am always happy, always desirous to perform this duty. Am I then in danger?"

The Empress embraced him, and asked:

"Do you love me now as ever? as in old time?"

"Love you? yes! How should I not love you? The day we saw one another for the first time, my heart said to me, There is one who is to be your guardian angel throughout life, and this prediction of my heart has been accomplished. You are weeping!" Her tears flowed apace, and she began to repeat the Lord's Prayer in a low tone. The Emperor followed the words, and added, in a firm voice, when the Empress pronounced, "Thy will be done!" "Yes, in all things and always."

The Emperor understanding all, turned a firm and scrutinizing look on the physician, saying:

"Tell me, then, what is it? Am I dying?"

Choked with emotion, the physician said: "Yes, sire!"

After a brief silence, the Emperor inquired:

"What have you discovered in me with your stethescope? Abscesses?"

"No; but the commencement of paralysis of the lung."

"And you have had the courage, thereupon, to pronounce my sentence—to condemn me definitively to death?"

His physician reminded him that he acted in accordance with a promise exacted by the Emperor a year and a half before, when the Emperor said to him:

"I require of you to tell me the whole truth, and in time, when you see that there is need."

The Emperor listened with calm attention, and replied: "I thank you."

At the Emperor's request, the family were called in. After prayers, the Emperor said, "I pray the Lord to receive me in his arms," and partook of the Sacrament with the utmost calmness and devout fervor. After having first rendered to God the things that are God's, Nicholas next turned his attention to the affairs of his vast empire. He ordered telegrams to the chief cities to say, "The Emperor is dying," adding, "The Emperor bids adieu to Moscow." He ordered his funeral to be conducted with as little expense as possible, as it would fall ultimately on his people.

He spoke or sent kind words of remembrance to every member of his immediate household, not forgetting a child or a grandchild. But his tenderest and most constant attention was centered on her who had so long traversed with him the vicissitudes of life and empire. Addressing her, and pointing to his children present, he said: "You must live for them." And to his children: "Live always, as now, in the closest union of family affection."

A courier arrived from the Crimea, bringing letters and dispatches from his sons, the Grand Dukes Nicholas and Michael. "Are they well?" inquired the Emperor. "The rest is nothing to me now. I belong wholly to God."

He then asked his physician with a smile:

"When do you mean to let me go?"

"Not yet."

"Shall I not wander, or become insensible?"

"I hope, sire, that all will pass quietly."

Then embracing his son and successor, the present Emperor, he said:

"I could have wished to have taken upon myself all that is difficult and painful, and to have left you an empire at peace, happy and flourishing. Providence has ordered it otherwise. Now I go to pray in the other world for Russia, and for you, who are, after Russia, that which I have most loved in this world."

When, having no longer strength to speak, he made a gesture between his almoner and his successor and the Empress, with what meaning must be left to conjecture; most likely it was: "Stand by one another." From that time he held their hands in his, pressing them from moment to moment, until he ceased to breathe.

From the last will of Nicholas the First, every affectionate family may learn a practical lesson of great value, in all cases, showing what a close affection existed in the royal household. It was written nearly eleven years before, to wit, on Ascension Day, May 4, 1844.

The great Autocrat, whose word and will were law to sixty million souls, seems, in the contemplation of death, although in perfect health, to have divested himself of all factitious greatness, and to have felt he was but a man; so he entitled the document his "Last Wishes." After the invocation to God the Father, God the Son, and God the Holy Ghost, come these words:

"In June, 1831, at the time of the breaking out of the cholera, I wrote down hastily my last wishes. The Lord in his mercy has not only been pleased to preserve my family, but further to permit that, since then, it should be considerably increased. These happy circumstances are of a nature to change, in part, my intentions. I desire that my wife be left in possession, for her use, of her apartments in the Winter Palace, of the Palace of Jelagin, and in the New Palace. Further, although by right of inheritance the Amtchkoff Palace belongs to my eldest son, I leave it to my wife, to enjoy it during her life, if she pleases.

"I adjure and beseech my children and grand-children to love and honor their mother, to be attentive to her comfort, to anticipate all her wishes, and to strive to console her in her old age by their affection and devotion. They ought never to undertake any thing of importance without having first asked her advice and her motherly blessing. Such of my children as are under age are to remain entirely dependent upon her will until they attain their majority."

It is delightful to contemplate that after the Emperor had provided for his own blood and kindred, he turns himself with an affectionate remembrance to his servants and the teachers and guides of his youth, and of "my invalids," as he calls them. And seeming at the moment of writing the words to regard himself as no longer on the throne, he says: "I beg the Emperor to be so good as to take care of my old invalids. I wish that they should keep during their lives, in the Imperial House, the places which they filled during my time, unless it please my son to find them any thing better.

"Since my childhood, two friends and companions have remained at my side, and their friendship for me has been invariably the same. I loved the General Aid-de-camp, Adlerbergh, as my brother, and I hope to have in him a friend to the end of my life. His sister, Julia, has educated my three daughters like a good and tender parent. I bequeath to them a pension of fifteen thousand silver rubles, besides the pension they have already. I thank them once more, for the last time, for their brotherly affection.

"I thank Count Benkendorf and Count Orloff for their unchangeable friendship, for their constant attachment to my person, and for the activity and zeal which they have shown in the execution of my orders."

Speaking of his sister, Maria Paulovina, he thus expresses himself:

"I have ever professed, from my childhood, the most sincere gratitude for all her goodness to me. Later in life her friendship has been still more precious to me. In her I put my greatest trust. I respected her as a mother, and confessed to her all my feelings, all the thoughts of my heart. I declare to her, for the last time, my tender gratitude for all the happy moments I have passed with her.

"I beg the Emperor and all my family to love and honor my brother and sincere friend, Michael Paulovitch. He is a pattern of that affection with which a brother ought to serve his brother. I beg him not to cease to lend his good advice to my children, whom I recommend always to his good-will. I conjure my children to love their Emperor with all their love, to honor him and serve him faithfully, indefatigably, and unresistingly, to the last drop of their blood, to their last breath; to remember that they ought to be examples to the rest of his subjects, as they are his first subjects.

"I am convinced that my son, the Emperor Alexander, will be always towards his mother a dutiful son, as he has been to me. This duty will become more sacred from the moment she is left alone. In her bereavement it is right that she be comforted by the love and tender attachment of her children and grand-children. In his relations with his brothers, the Emperor my son should strive to unite that indulgence which their youth requires, with necessary firmness, as a father of a family, and never tolerate any misunderstanding or family quarrel, nor any thing which may become prejudicial to them or the state.

"I thank all who have loved and served me. I forgive all who have hated me, and I pray all whom I may have unintentionally offended to forgive me. I have been a man, and subject to a man's weaknesses. I have striven to correct myself in what I perceived to be amiss in me; I have succeeded in doing this in some respects; I have not succeeded in others. I pray all, with all my heart, to forgive me.

"I die with my heart full of gratitude for all the good that it has pleased God to grant me in this transitory life, full of ardent love for our glorious Russia, which I have served according to my ability, with faith and sincerity. It is in thee, O Lord! that we trust—so may we never be put to confusion!"

A year later he adds:

"On the 29th of July, 1844, it pleased God to take to himself our beloved daughter Alexandra. We endure, without murmuring, bowing before the decree of the Lord, this terrible blow, being fully assured, that if such was the will of God, it was for the good of our dear daughter; and full of hope that she is now more happy in heaven than she could have been here on earth."

With such a view of the inner life of the great Nicholas the First of Russia, it can scarcely be doubted that he was a Christian man; while the clear exhibitions of his forgiving nature towards his enemies, and of his tender affection to every member of his family, his children, his grand-children, his brother, his sister, his teachers, his servants, his counselors and friends, and more than all, that tender love which he manifested towards his wife, the unlimited confidence which he had in her wisdom and sound judgment, and his steady solicitude that every effort should be made for her comfort and happiness when he was gone, all these show that Nicholas of Russia was a noble man; noble by nature as well as by birth; and that for the things named he is worthy to be held up for all time to the admiration of the world, as a bright example to succeeding generations.

Let the great and wise Emperor's views as to the family relation make an ineffaceable impression on every father's heart in making his will.

First: Keep the children as long as possible entirely dependent on the will of the mother.

Second: Inculcate, persuade, command, and adjure, that the members of the family are to stand by one another faithfully, encouragingly, and affectionately, under all conceivable circumstances to the very end, when not incompatible with clearly higher duties; to stand up for one another, in case of need, against a universe besides: they who do it not, be they men or women, parents or children, brothers or sisters, husbands or wives, are unworthy of the name or relationship.

The want of this loving fidelity to one's own blood has broken many an innocent and striving heart; has caused life-long animosities in large family connections, theretofore loving and united; and has deluged nations in blood.

